# Internal Jugular Vein Fenestration: An Intraoperative Finding Without a Radiological Clue

**DOI:** 10.7759/cureus.21166

**Published:** 2022-01-12

**Authors:** Youssef Aladham, Sheikh Muktadir Bin Momin, Omar Ahmed, Samuel Jackson

**Affiliations:** 1 Otolaryngology, East Kent Hospitals University NHS Foundation Trust, Ashford, GBR; 2 Otolaryngology, Bristol Royal Infirmary, Bristol, GBR

**Keywords:** neck dissection, split, duplication, fenestration, anatomical anomalies, internal jugular vein

## Abstract

A comprehensive understanding of the anatomical variations of the internal jugular vein (IJV) is essential to prevent inadvertent injuries during neck procedures, particularly neck dissection. In addition, its relationship with the spinal accessory nerve in the upper part of the neck is relatively variable. IJV fenestration refers to bifurcation of the vein with reunion proximal to the subclavian vein, whereas IJV duplication refers to continued branching till joining the subclavian vein separately. We report a case of a fenestrated IJV identified intraoperatively with the spinal accessory nerve passing laterally to both divisions.

## Introduction

Neck dissection is a frequent procedure performed in patients with head and neck malignancy. Due to the significant morbidity associated with their injuries, anatomical and functional preservation of the internal jugular vein (IJV), spinal accessory nerve (SAN), and sternocleidomastoid muscle, when oncologically appropriate, has gained more attention. The SAN most commonly crosses superficial to the upper part of the IJV, but its passage medial to the vein has also been reported in up to 3% of patients. Rarely, the SAN passes through the IJV posing a risk of injury to the vein near the skull base, where control of bleeding is surgically challenging [1]. Variations in the anatomy of the IJV are rare, but a few reports described duplication of the vein shortly after exiting the skull base till joining the subclavian vein separately. Fenestration refers to branching of the vein with distal re-joining lower in the neck. Prades et al. reported three cases of IJV malformation (duplication or fenestration) in 750 neck dissection cases with a prevalence of 0.4% [2]. In the fenestration variant, the SAN was commonly found passing through the window [3].

## Case presentation

A 69-year-old male patient presented to the otolaryngology clinic via the rapid access pathway with a painless left neck lump that had existed for one month with no other head and neck or upper aerodigestive symptoms. The patient was otherwise healthy and a lifelong smoker. Examination revealed an approximately 4 cm left level II mass that was soft, mobile, and cystic. Oral cavity examination and office pharyngolaryngoscopy were unremarkable.

Radiological workup, including neck ultrasound and magnetic resonance imaging (MRI), revealed a solitary left cystic level II mass with a solid component, suspicious of nodal malignancy. No other cervical lymphadenopathy or evidence of a primary was noted. Ultrasound-guided fine-needle aspiration was repeatedly inconclusive. A positron emission tomography (PET) scan showed intense metabolic activity in the mass and the rest of the examination was normal. A multidisciplinary team (MDT) recommended neck dissection of left level II and III territory along with panendoscopy.

**Figure 1:**
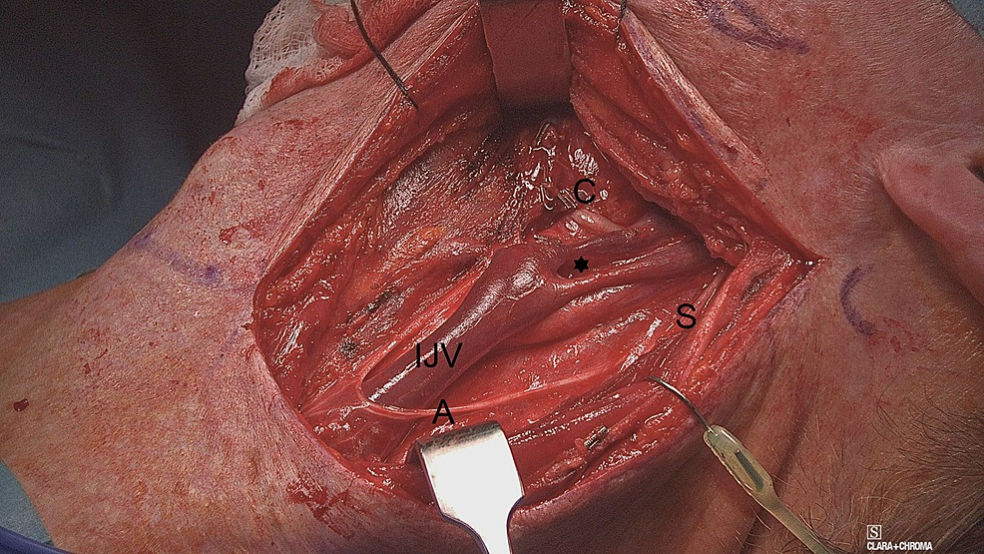
Fenestration of the left IJV Intraoperative appearance of the left neck with fenestrated IJV after neck dissection was completed. The common facial vein (C) is clipped and divided. S, spinal accessory nerve; A, ansa cervicalis. The asterisk refers to the window (fenestration). IJV, internal jugular vein.

Intra-operatively, neck dissection proceeded smoothly, and the SAN was identified and secured. The IJV was found to be splitting from near the skull base to 1 cm above the left of carotid bifurcation (Figure 1). The spinal accessory nerve was crossing lateral to both divisions of the IJV. The common facial vein was clipped and divided as it was encased by the mass. It was the point of reunion of the IJV divisions where the common facial vein joined the IJV. Sternocleidomastoid muscle, IJV, and spinal accessory nerve were all preserved. Panendoscopy failed to demonstrate abnormalities.

**Figure 2:**
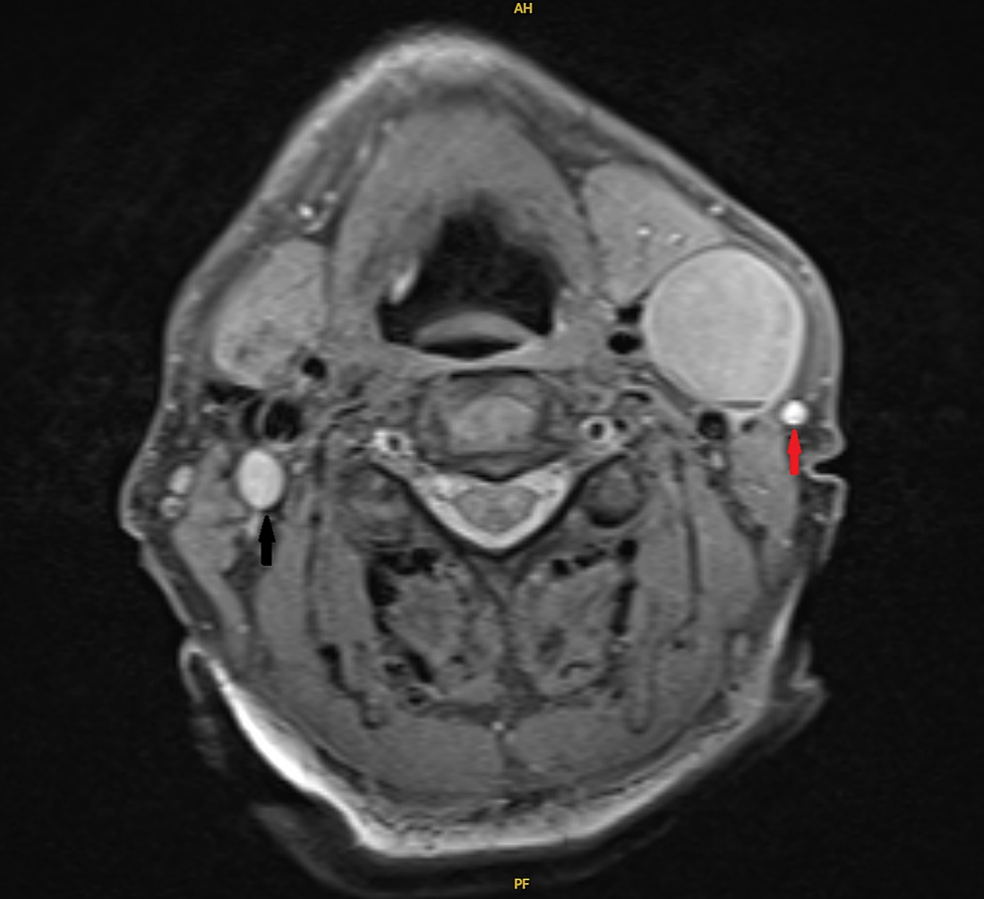
Contrast-enhanced MRI. A contrast-enhanced MRI, axial view, showing completely non-visualized left IJV that is compressed by the mass. The right IJV (black arrow) and left external jugular vein (red arrow) can be seen. IJV, internal jugular vein.

Retrospective reassessment of the contrast-enhanced MRI revealed that the IJV was compressed by the mass and totally collapsed at that level, hindering preoperative radiological identification of the variation (Figure 2).

Histologic examination of the neck dissection specimen confirmed P16-positive squamous cell carcinoma (SCC) of the neck. Tran-oral robotic tongue base mucosectomy confirmed a microscopic tongue base primary.

## Discussion

Injury of the IJV may occur during prophylactic or therapeutic neck dissection, as well as during central venous catheter placement in the neck. Besides associated morbidity from its ligation or occlusion, control of bleeding, especially near the skull base, is surgically difficult and may require drilling of the mastoid and vascular control at the level of sigmoid sinus [4]. To avoid that, an early step during neck dissection is to control the proximal end of the vein (near the skull base) followed by control of the distal end. Failure to recognize unusual anatomical variations of the veins may lead to inadvertent injury and hard-to-control bleeding. When the IJV is duplicated or fenestrated in the upper neck, a surgeon may recognize one branch as the IJV, and continue dissection unmindful of the presence of another branch resulting in its injury and consequent bleeding [2].

IJV anomalies can be visualized preoperatively by contrast-enhanced imaging such as computed tomography (CT) or MRI of the neck performed for evaluation of the primary neck disease [5]. That said, evidence from the literature demonstrates that it is most commonly encountered intraoperatively without prior anticipation, reflecting the lack of attention paid to assessing the venous system in the neck. Moreover, should there be compression of the anomalous IJV by the cervical lymphadenopathy, it is merely not possible to visualize the IVJ at all on contrast imaging due to the collapsible nature of the venous wall [6]. In our case, the IJV was compressed by the large lymph node halting its identification on contrast-enhanced MRI.

It is worth mentioning that in our case, the carotid vessels were located posteromedial to both branches of the IJV, and the common facial vein (that had to be divided) drained into the anteromedial branch before the re-joining point. Additionally, the SAN maintained a superficial position to both branches of the IJV.

## Conclusions

Anomalous IJV could present as an intraoperative finding without prior anticipation by contrast-enhanced cross-sectional imaging. The spinal accessory nerve may cross superficially to both branches of a fenestrated IJV and care must be taken to avoid injury to both.
